# GSEL: a fast, flexible python package for detecting signatures of diverse evolutionary forces on genomic regions

**DOI:** 10.1093/bioinformatics/btad037

**Published:** 2023-01-19

**Authors:** Abin Abraham, Abigail L Labella, Mary Lauren Benton, Antonis Rokas, John A Capra

**Affiliations:** Division of General Pediatrics, Children’s Hospital of Philadelphia, Philadelphia, PA 19104, USA; Department of Biological Sciences, Vanderbilt University, Nashville, TN 37232, USA; Evolutionary Studies Initiative, Vanderbilt University, Nashville, TN 37232, USA; Department of Bioinformatics and Genomics, University of North Carolina, Charlotte, NC 28223, USA; North Carolina Research Center, Kannapolis, NC 28081, USA; North Carolina Research Center, Kannapolis, NC 28081, USA; Department of Biological Sciences, Vanderbilt University, Nashville, TN 37232, USA; Evolutionary Studies Initiative, Vanderbilt University, Nashville, TN 37232, USA; Department of Computer Science, Baylor University, Waco, TX 76706, USA; Department of Biomedical Informatics, Vanderbilt University School of Medicine, Nashville, TN 37232, USA; Vanderbilt Genetics Institute, Vanderbilt University, Nashville, TN 37232, USA; Department of Epidemiology and Biostatistics, Bakar Computational Health Sciences Institute, University of California, San Francisco, CA 94143, USA

## Abstract

**Summary:**

GSEL is a computational framework for calculating the enrichment of signatures of diverse evolutionary forces in a set of genomic regions. GSEL can flexibly integrate any sequence-based evolutionary metric and analyze sets of human genomic regions identified by genome-wide assays (e.g. GWAS, eQTL, *-seq). The core of GSEL’s approach is the generation of empirical null distributions tailored to the allele frequency and linkage disequilibrium structure of the regions of interest. We illustrate the application of GSEL to variants identified from a GWAS of body mass index, a highly polygenic trait.

**Availability and implementation:**

GSEL is implemented as a fast, flexible and user-friendly python package. It is available with demonstration data at https://github.com/abraham-abin13/gsel_vec.

**Supplementary information:**

Supplementary data are available at *Bioinformatics* online.

## 1 Motivation

Over the last 15 years (Claussnitzer et al., 2020; Loos, 2020), the proliferation of low-cost genotyping and genome sequencing has enabled the discovery of millions of associations between genotypes and phenotypes, at both the molecular and organism scales (Canela-Xandri *et al.*, 2017; Watanabe *et al.*, 2019). The determination of patterns of DNA sequence variation for thousands of diverse individuals has also enabled development of methods for quantifying signatures of different evolutionary forces, including diverse modes of natural selection (e.g. negative, positive and balancing selection over different time scales) (Fan *et al.*, 2016; Pritchard *et al.*, 2010; Rees *et al.*, 2020; Vitti *et al.*, 2013). Understanding the history of evolutionary forces on loci associated with a trait offers powerful insights that can guide prioritization of variants for downstream analyses and answer fundamental questions about the evolution of traits (Benton *et al.*, 2021; Guo *et al.*, 2018; LaBella *et al.*, 2020; Sella and Barton, 2015). However, rigorously evaluating whether observed evolutionary patterns in regions of interest differ from expected values remains challenging, because genomic features such as minor allele frequency (MAF) and linkage disequilibrium (LD) influence statistical power to detect both genome-wide associations and evolutionary signatures.

**Fig. 1.**
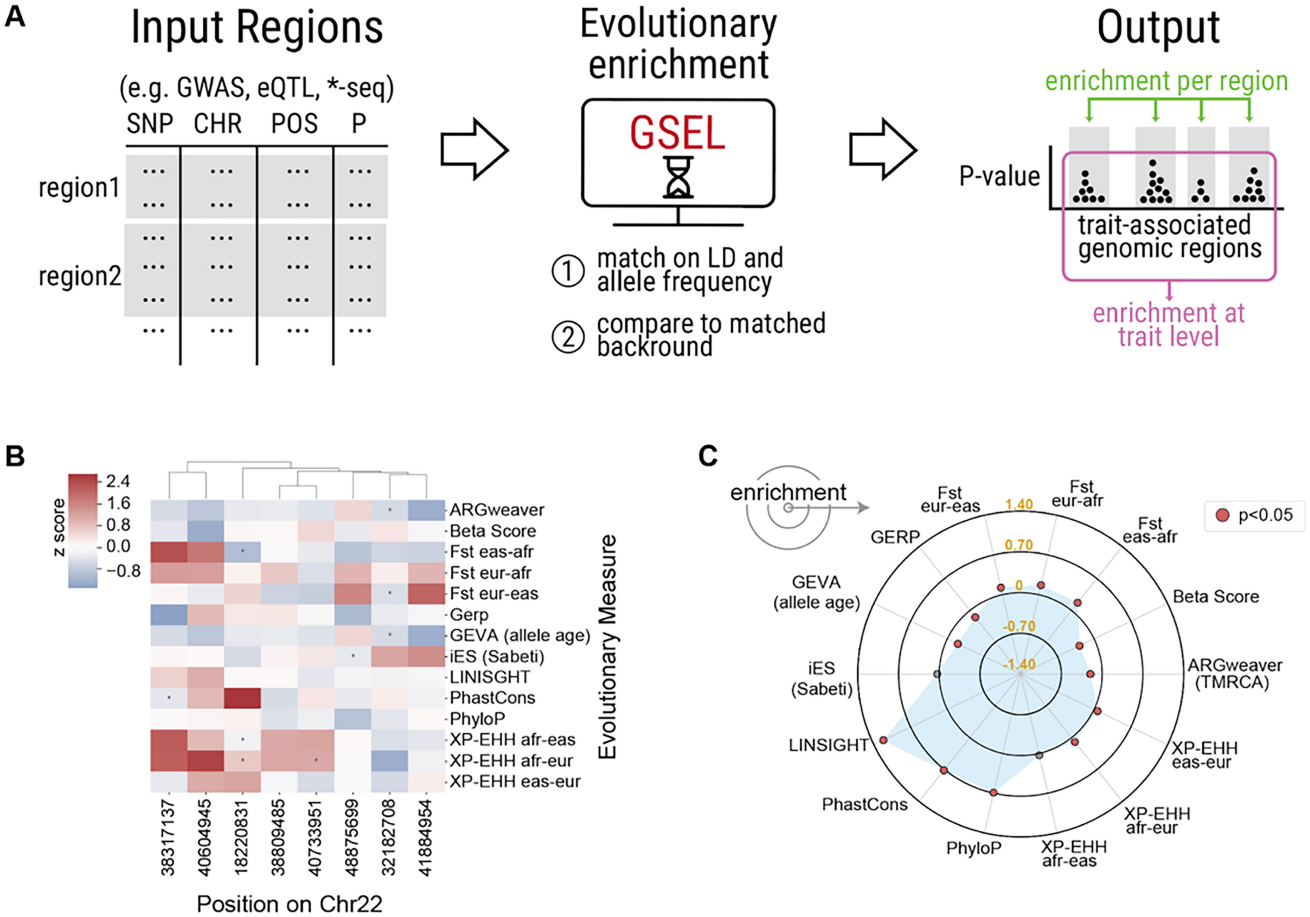
GSEL detects evolutionary enrichments from genomic region sets. (**A**) Using GWAS summary statistics as an example input, GSEL outputs enrichment for evolutionary measures at the region- and trait-level based on a matched background distribution. (**B**) After applying GSEL on a GWAS of body mass index, the region level enrichments for chromosome 22 are quantified by a z-score (color bar) for trait-associated regions (columns) and 14 evolutionary measures (rows). (**C**) Trait-level enrichments for the body mass index GWAS are visualized in a radar plot with each spoke representing an evolutionary measure and enrichment measured along the radial axis. Evolutionary measures include F_ST_, iES, XP-EHH (allelic differentiation within human populations), Beta Score (balancing selection), allele age (time to most recent common ancestor, TMRCA, from ARGweaver or GEVA), and PhyloP, PhastCons, LINSIGHT, GERP (conserved/accelerated substitution rates between species)

Here, we describe GSEL, a computational framework that calculates region- and trait-level enrichments for diverse evolutionary measures (Fig. 1). GSEL builds appropriate null distributions for each region and trait conditioned on genomic features that influence power and ascertainment.

GSEL provides a simple command line interface that seamlessly integrates disparate computational steps. GSEL’s built-in parallelization and vectorization enable rapid processing of large numbers of sets (each of which may contain many genomic regions), even when generating empirical backgrounds based on thousands of permutations each with thousands of control regions. GSEL currently includes 14 diverse measures of different evolutionary signatures by default, and it can additionally incorporate any evolutionary measure with genome-wide quantification. By integrating the wealth of available data on associations between genomic loci and phenotypes with methods for detecting genetic signatures of distinct evolutionary forces, GSEL enables characterization of the genome–phenome map through an evolutionary lens.

## 2 Usage

GSEL can be run from the command line by specifying a set of genomic regions with trait associations. GSEL can analyze any set of regions, but here we illustrate its application to regions identified by a genome-wide association study (GWAS). After installation, a test suite is provided to ensure the pipeline functions as expected.

## 3 Analysis methods

GSEL computes region- and set-level enrichments for evolutionary measures by comparing observed values to empirically generated null distributions. GSEL begins by identifying independent LD blocks among the input regions (e.g. independent trait-associated regions from GWAS) using the ‘--clump’ flag in PLINK (Chang *et al.*, 2015). Each region is labeled by the single-nucleotide polymorphism (SNP) with the lowest *P*-value, which we refer to as the index SNP. Next, GSEL randomly selects 5000 SNPs for each index SNP matching on MAF (±5%) and LD structure (number of SNPs with *r*^2^ ≥ 0.9) and expands to include control SNPs based on LD. By default, GSEL considers 1000 Genomes Phase 3 data from the European super-population and uses the algorithm and default parameters in SNPSNAP (Pers *et al.*, 2015); however, all of these references and thresholds can be customized. Together, the matched LD expanded control SNPs compose a matched region.

For a given evolutionary measure, the most extreme values across control SNPs in each matched region form a background distribution. GSEL then quantifies enrichment as a z-score based on the extreme value from the trait-associated region and the background distribution. An empirical *P*-value is obtained by comparing the number of matched regions with a value equal to or more extreme than the observed trait value. Multiple testing correction over all trait-associated regions is performed using the Benjamini–Hochberg method for false discovery rate control.

GSEL also calculates set-level enrichments (e.g. across all regions associated with a trait). For an evolutionary measure, the set-level average is calculated based on a summary statistic computed across the extreme values at each region (e.g. mean or max). To generate a set-wide background distribution, GSEL generates matched sets (default: 5000) from the matched SNPs, where each set has one matched region for each region in the input set. For every set, GSEL calculates the summary statistic across all the extreme values at each matched region. Evolutionary enrichment at the region-level is defined as the region-level statistic subtracted from the background statistic and divided by the genome-wide standard deviation for that evolutionary measure. GSEL also calculates an empirical *P*-value using the background distribution as described for the region-level *P*-value.

## 4 Outputs and interpretation

The total run time of GSEL scales with the number of input regions. For large inputs, a user can partition regions into an arbitrary number of independent bins. For example, each chromosome can be analyzed simultaneously, and the results then combined to compute enrichments. To benchmark GSEL performance (Supplementary Methods), we applied GSEL to 47 GWASs of human traits (Loh *et* al., 2015). Body height, which had the largest number trait-associated regions (*n* = 6682), required 43 GB of memory and took 8:54:30 (h:min:s) and 4:29:18 for per region and trait-level analyses respectively on an Intel(R) Xenon(R) CPU ES-2420 at 1.90 and 2.40 GHz. Detailed benchmarks are in Supplementary Table S1.

In addition to producing tabular summaries, GSEL produces publication-ready heatmaps and radar plots for region- and set-level enrichments. The region-level plots (Fig. 1B) are labeled according to index SNPs (columns) and z-scores for evolutionary measures (rows). For example, for a body mass index GWAS, chromosome 22 contains multiple regions with strong signals of potential recent positive selection (XP-EHH) and local adaptation (F_ST_). In the set-level radar plot (Fig. 1C), each evolutionary measure is a spoke, and each ring represents the enrichment. We observe strong enrichment for signatures of negative selection (e.g. LINSIGHT, PhastCons, PhyloP).

## 5 Conclusion

GSEL is a fast, flexible and user-friendly computational framework for calculating enrichment for evolutionary signatures in region sets of interest. GSEL requires only genomic coordinates as input. Even for input sets with thousands of regions, GSEL can efficiently compute enrichment in a few hours on a single computer. GSEL can easily be deployed on high-performance computing systems with PLINK and python packages managed through Anaconda. For example, we applied GSEL on over 900 GWASs of human traits and found a mosaic pattern of selection on trait-associated genomic regions (Abraham *et al.*, 2022). Finally, new evolutionary measures and non-European datasets can be analyzed with minimal modifications (see GitHub repository for instructions). GSEL can be applied after any genomic analysis to provide evolutionary context for hypothesis generation and downstream analyses.

## Supplementary Material

btad037_Supplementary_Data

## Data Availability

All code and data are available at https://github.com/abraham-abin13/gsel_vec.
