# Supplementary material for: GSEL: a fast, flexible python package for detecting signatures of diverse evolutionary forces on genomic regions
Source: Bioinformatics. 2023 Jan 19;39(1):btad037. doi: 10.1093/bioinformatics/btad037 (PMC9879724; doi:10.1093/bioinformatics/btad037)
Supplement: btad037_Supplementary_Data [file btad037_supplementary_data.docx]

**Supplemental Information**

Benchmarking Performance

To evaluate GSEL performance, we selected a robust dataset of 47 GWAS performed by Loh et al[[16]](https://app.readcube.com/library/3db2c3cd-b81f-481e-85eb-cd7b13db418e/all?uuid=9129696990186729&item_ids=3db2c3cd-b81f-481e-85eb-cd7b13db418e:57282963-e44e-473d-a7f8-1d7b9cab76b1) in a large European cohort (n~450,000 individuals). The GWAS summary statistics were calculated using a Bayesian mixed modeling strategy over 9.6 million imputed SNPs and filtered based on the genome-wide significance threshold (P<5x10^-8^). We downloaded the summary statistics from <https://alkesgroup.broadinstitute.org/UKBB/>.

In this dataset, we ran GSEL in two steps. For each trait, we split summary statistics by chromosome and calculated the enrichment per genome-wide significant region. After that analysis was complete, we calculated the enrichment at the trait level. See the GitHub repository section titled “Running one chromosome at a time” for detailed methods.

All analyses were performed on the Advanced Computing Center for Research and Education computing cluster at Vanderbilt University using batched array job submissions. For each job submitted, we used a 64-bit Intel® Xenon® CPU E5-2420 at 1.90GHz or at 2.40GHz with 24 total compute cores. The total memory required and run time is reported in Supplementary Table 1.

|  | # of Regions | Per Region Analysis | | | | Trait-level Analysis | |  |
| --- | --- | --- | --- | --- | --- | --- | --- | --- |
|  |  | Memory (GB) | | Run Time (H:M:S) | | Memory (GB) | Run Time (H:M:S) | |
| Trait |  | Min | Max | Min | Max |  |  | |
| body_heightz | 6,682 | 17.5 | 30.5 | 0:40:05 | 8:54:30 | 43.0 | 4:29:18 | |
| blood_mean_platelet_vol | 4,133 | 16.2 | 29.7 | 0:21:05 | 5:59:52 | 23.2 | 3:01:06 | |
| blood_platelet_count | 3,055 | 15.7 | 28.1 | 0:25:42 | 4:08:44 | 20.7 | 2:35:38 | |
| bmd_heel_tscorez | 2,991 | 15.9 | 27.2 | 0:33:56 | 4:05:45 | 20.6 | 2:31:28 | |
| impedance_metabo_rate | 2,265 | 12.7 | 25.9 | 0:18:53 | 2:51:35 | 19.8 | 2:30:24 | |
| blood_platelet _width | 2,194 | 15.4 | 26.6 | 0:20:44 | 4:04:04 | 14.6 | 1:42:39 | |
| blood_MCH | 2,192 | 13.6 | 26.6 | 0:24:27 | 3:32:57 | 15.5 | 2:02:49 | |
| blood_sphered_cell_vol | 1,899 | 12.9 | 26.6 | 0:20:04 | 2:47:46 | 14.2 | 1:41:21 | |
| blood_monocyte_count | 1,897 | 12.2 | 26.5 | 0:19:21 | 2:51:02 | 13.6 | 1:39:25 | |
| pigment_hair | 1,877 | 7.2 | 25 | 0:14:14 | 2:23:19 | 10.8 | 1:14:28 | |
| blood_red_count | 1,842 | 14.6 | 24.9 | 0:29:07 | 2:33:46 | 15.2 | 1:36:24 | |
| blood_eosinophil_count | 1,693 | 13.6 | 24.9 | 0:28:36 | 1:58:16 | 12.6 | 1:40:58 | |
| blood_rbc_distrib_width | 1,635 | 14.2 | 25.4 | 0:24:04 | 1:53:25 | 12.1 | 1:30:58 | |
| blood _retic | 1,578 | 12.2 | 25.8 | 0:18:20 | 2:04:59 | 12.4 | 1:31:24 | |
| blood_lymphocyte_count | 1,480 | 12.2 | 24.6 | 0:18:13 | 1:44:48 | 11.9 | 1:31:53 | |
| body_bmiz | 1,345 | 12.8 | 24.4 | 0:19:24 | 1:58:24 | 13.5 | 1:27:34 | |
| blood_white_count | 1,287 | 9.9 | 23.2 | 0:20:44 | 1:48:29 | 11.8 | 1:21:37 | |
| lung_fev1fvczsmoke | 1,227 | 11.6 | 24.2 | 0:20:02 | 1:42:20 | 11.8 | 1:28:30 | |
| bp_diastolicadjmedz | 1,079 | 5.5 | 22.7 | 0:15:09 | 1:27:23 | 10.9 | 1:06:57 | |
| pigment_hair_blonde | 1,042 | 3.1 | 23 | 0:12:36 | 1:26:01 | 7.7 | 0:47:22 | |
| bp_systolicadjmedz | 1,015 | 12.5 | 22.3 | 0:17:37 | 1:32:19 | 12.0 | 1:19:49 | |
| pigment_hair_darkbrown | 1,010 | 0 | 25.3 | 0:00:31 | 1:22:50 | 6.7 | 0:38:53 | |
| body_whradjbmiz | 860 | 12.6 | 23 | 0:18:03 | 1:10:59 | 8.3 | 1:01:17 | |
| pigment_skin | 852 | 3.8 | 23 | 0:13:38 | 1:41:42 | 5.3 | 0:29:49 | |
| body_balding1 | 790 | 4 | 22.2 | 0:15:50 | 1:21:49 | 7.1 | 0:38:03 | |
| lung_fvczsmoke | 743 | 5.6 | 19.7 | 0:16:46 | 1:15:34 | 10.1 | 1:06:48 | |
| body_balding4 | 721 | 3.5 | 20.4 | 0:15:25 | 1:15:40 | 6.7 | 0:34:58 | |
| repro_menarche_age | 464 | 7.1 | 21.5 | 0:14:19 | 0:52:25 | 6.8 | 0:36:50 | |
| disease_cardiovascular | 381 | 3.1 | 17.2 | 0:14:23 | 0:40:14 | 5.6 | 0:33:32 | |
| cov_edu_college | 340 | 0.1 | 22 | 0:01:13 | 0:45:56 | 5.3 | 0:27:38 | |
| cov_edu_years | 334 | 3.5 | 20.8 | 0:13:44 | 0:49:37 | 5.2 | 0:28:07 | |
| pigment_sunburn | 310 | 0 | 19.9 | 0:00:24 | 1:07:52 | 2.7 | 0:11:37 | |
| disease_allergy_eczema | 300 | 3.8 | 20.1 | 0:14:05 | 0:36:47 | 4.3 | 0:21:20 | |
| disease_hypothyroidism | 219 | 3.2 | 21 | 0:14:18 | 0:32:59 | 3.1 | 0:15:55 | |
| disease_asthma | 216 | 2.9 | 22.3 | 0:13:32 | 0:32:50 | 3.3 | 0:18:15 | |
| disease_hi_chol | 213 | 0 | 21.2 | 0:00:28 | 0:40:00 | 2.5 | 0:09:46 | |
| other_morningperson | 192 | 0 | 19.8 | 0:00:28 | 0:33:19 | 3.7 | 0:18:41 | |
| cov_smoking_status | 192 | 3.4 | 19.7 | 0:13:49 | 0:37:32 | 3.9 | 0:20:08 | |
| disease_respiratory_ent | 161 | 0 | 20.2 | 0:00:31 | 0:24:25 | 3.0 | 0:15:29 | |
| mental_neuroticism | 152 | 0 | 20.6 | 0:00:36 | 0:29:01 | 3.7 | 0:17:21 | |
| disease_t2d | 107 | 0 | 19.8 | 0:00:25 | 0:30:13 | 2.0 | 0:07:39 | |
| repro_menopause_age | 104 | 0 | 17.3 | 0:00:28 | 0:21:55 | 2.7 | 0:09:27 | |
| disease_aid_all | 101 | 3.1 | 19.2 | 0:13:53 | 0:21:50 | 2.4 | 0:08:17 | |
| pigment_tanning | 35 | 0 | 19 | 0:00:14 | 0:24:49 | 1.8 | 0:04:49 | |
| disease_dermatology | 28 | 0 | 14.2 | 0:00:22 | 0:20:13 | 0.9 | 0:03:32 | |
| repro_childreneverborn | 11 | 0 | 5.1 | 0:00:27 | 0:16:13 | 0.8 | 0:05:17 | |
| disease_aid_sure | 10 | 0 | 4.5 | 0:00:21 | 0:16:02 | 0.2 | 0:01:56 | |

**Supplemental Table 1: Benchmarking of GSEL runtime and memory use. We ran GSEL on** 47 GWAS studies of complex human traits and computed benchmark statistics. For each GWAS, we report the trait name (“Trait”) and the number of genome-wide significant regions (GWAS P-value < 5x10^-8^, “# of Regions”). Analysis was conducted in two steps. First, we calculated the evolutionary enrichment for each genome wide significant region (“Per Region Analysis”). Then we calculated the trait-wide evolutionary enrichment (“Trait-level Analysis”). The memory in gigabytes (“Memory (GB)” and run time (“Run Time (H:M:S)”) in hours:minutes:seconds are reported for each trait. Since the per region analyses was on each chromosome in parallel, we report the minimum (“Min”) and maximum (“Max”) across chromosomes 1-22.
